# Can automation and artificial intelligence reduce echocardiography scan time and ultrasound system interaction?

**DOI:** 10.1186/s44156-025-00077-0

**Published:** 2025-06-16

**Authors:** Kylie J. Hollitt, Steven Milanese, Majo Joseph, Rebecca Perry

**Affiliations:** 1 Allied Health and Human Performance, University of South Australia, GPO Box 2471, Adelaide, SA 5072 Australia; 2 School of Health Sciences, Physiotherapy, Swinburne University of Technology, Hawthorn, VIC Australia; 3 Department of Cardiology, Flinders Medical Centre, Southern Adelaide Local Health Network, Adelaide, SA Australia; 4 School of Medicine, Flinders University, Bedford Park, South Australia Australia

**Keywords:** Artificial intelligence, Automation, Transthoracic echocardiography, Ergonomics

## Abstract

**Background:**

The number of patients referred for and requiring a transthoracic echocardiogram (TTE) has increased over the years resulting in more cardiac sonographers reporting work related musculoskeletal pain. We sought to determine if a scanning protocol that replaced conventional workflows with advanced technologies such as multiplane imaging, artificial intelligence (AI) and automation could be used to optimise conventional workflows and potentially reduce ergonomic risk for cardiac sonographers. The aim was to assess whether this alternate protocol could reduce active scanning time as well as interaction with the ultrasound machine compared to a standard echocardiogram without a reduction in image quality and interpretability.

**Method and results:**

Volunteer participants were recruited for a study that comprised of two TTE’s with separate protocols. Both were clinically complete, but Protocol A combined automation, AI assisted acquisition and measurement, simultaneous and multiplane imaging whilst Protocol B reflected a standard scanning protocol without these additional technologies. Keystrokes were significantly reduced with the advanced protocol as compared to the typical protocol (230.9 ± 24.2 vs. 502.8 ± 56.2; difference 271.9 ± 61.3, *p* < 0.001). Furthermore, there was a reduction in scan time with protocol A compared to protocol B the standard TTE protocol (13.4 ± 2.3 min vs. 18.0 ± 2.6 min; difference 4.6 ± 2.9 min, *p* < 0.001) as well as a decrease of approximately 27% in the time the sonographers were required to reach beyond a neutral position on the ultrasound console.

**Conclusions:**

A TTE protocol that embraces modern technologies such as AI, automation, and multiplane imaging shows potential for a reduction in ultrasound keystrokes and scan time without a reduction in quality and interpretability. This may aid a reduction in ergonomic workload as compared to a standard TTE.

**Supplementary Information:**

The online version contains supplementary material available at 10.1186/s44156-025-00077-0.

## Background

A transthoracic echocardiogram (TTE) is one of the most widely used cardiac imaging modalities, allowing real time, rapid and non-invasive assessment of cardiac structures [1, 2]. The use of TTE has become a routine part of cardiac care, with recent focus being applied to advanced tools to assist in the reproducibility and standardization of TTE [3].

Workload, prolonged scanning time and the repetitive nature of sonography has been linked to the development of work-related musculoskeletal disorders (WRMSD) with increasing numbers of sonographers reporting pain from scanning [4, 5]. Four dimensional (4D, three-dimensional imaging with real time capture) and multiplane imaging show potential for optimising workflow by allowing simultaneous capture which may reduce keystrokes and scan time. Automation and Artificial Intelligence (AI) have also been proposed for use in TTE as ways to change workflow with suggestions that they may streamline image acquisition, reduce console keystrokes, examination time and cost [3]. TTE is well suited to AI due to the large volume of data acquired with each examination [2]. AI algorithms show promise for improved workflow and efficiency by utilising view recognition technology and automating measurements [6, 7].

The aim of the research study was to investigate whether a TTE protocol embracing modern technologies such as AI and automation, as well as multiplane imaging and semi-automated scan assisted protocols could reduce sonographer scan time and keystrokes as compared against a standard echocardiogram and therefore reduce the risk of WRMSD.

## Methods

### Study population

Volunteers were recruited using advertising within the University of South Australia. Exclusion criteria included age less than eighteen years and inability to provide written, informed consent. Volunteers were required to undergo two different TTE protocols at two separate time points, within two weeks of each other, at a similar time of day.

Participants were allocated to each sonographer based on the sonographer’s availability. If it was the participants first scan, the first protocol was determined by a coin flip immediately prior to scanning. On their second visit the protocol not previously undertaken was used. Demographic data such as date of birth, gender, height, weight and cardiac risk factors were collected. Height was assessed to the nearest centimetre using a portable stadiometer (Seca 213, Hamburg, Germany). Body mass was measured in minimal light clothing using portable digital weighing scales (Tanita Innerscan, Body Composition Monitor, BC-541). Body Surface Area (BSA) was calculated according to the formula of Dubois and Dubois as a ratio of weight (kilograms) to height (metres) [8].

Participants with prior cardiac disease were not excluded as varying cardiac pathology would help test the scan protocol. Convenience sampling was chosen as the project focused on scan time length, ultrasound system interactions, and not participant demographics. Sample size was calculated based on the average keystrokes in a full TTE study (approximately 500 ± 40) and an estimated decrease of 25% in keystrokes for an automated protocol. A sample size of 6 participants undergoing both protocols had 90% power to calculate a significant change at an alpha of 0.05, however, to ensure robustness of the study a sample size of 35 was used.

### Echocardiography/ image acquisition

The study comprised of two TTE protocols (A and B) that were performed by two experienced accredited medical sonographers using a commercially available ultrasound imaging system (204 Vivid E95 GE HealthCare, Horten, Netherlands) with a 4D capable phased-array transducer. Both sonographers were left-handed scanners with all console movements performed with the right hand.

Protocol A was designed to meet the American Society of Echocardiography (ASE) guidelines [9], but implemented commercially available, on cart automation, a semi-automated scan assistant protocol, AI, simultaneous colour doppler and multiplane imaging such as biplane and triplane imaging. Protocol B was a full, manually executed TTE study also according to the ASE guidelines [9] with all recommended measurements for a comprehensive assessment by TTE measured by the sonographer at the time of the scan without the use of automation or AI.

A specific machine protocol was created for protocol A using a scanning assistant program (Scan Assist Pro GE HealthCare, Horten, Netherlands) that directed users through a standardized step-by-step ultrasound examination using the image store button as a trigger to move to the next stage. Upon starting the chosen customised user defined protocol, the program utilised an automated exam script with preprogrammed imaging modes such as colour Doppler and biplane/ triplane (multiplane) imaging as well as imaging parameters such as depth, colour scale and zoom applied automatically to each step and updated with consecutive views. For steps that were pre-programmed with a mode that required user interaction (motion mode, continuous and pulse wave Doppler) the cursor automatically appeared on the 2D image to allow for alignment then the operator was required to initiate the correct mode with a button press. Specific measurements were built into individual steps of the protocol which were immediately available upon selection of the measure trigger removing the need to manually search and select the appropriate measurement [10]. Users paused the scanning assistant to incorporate further images in the case of pathological findings.

**Fig. 1:**
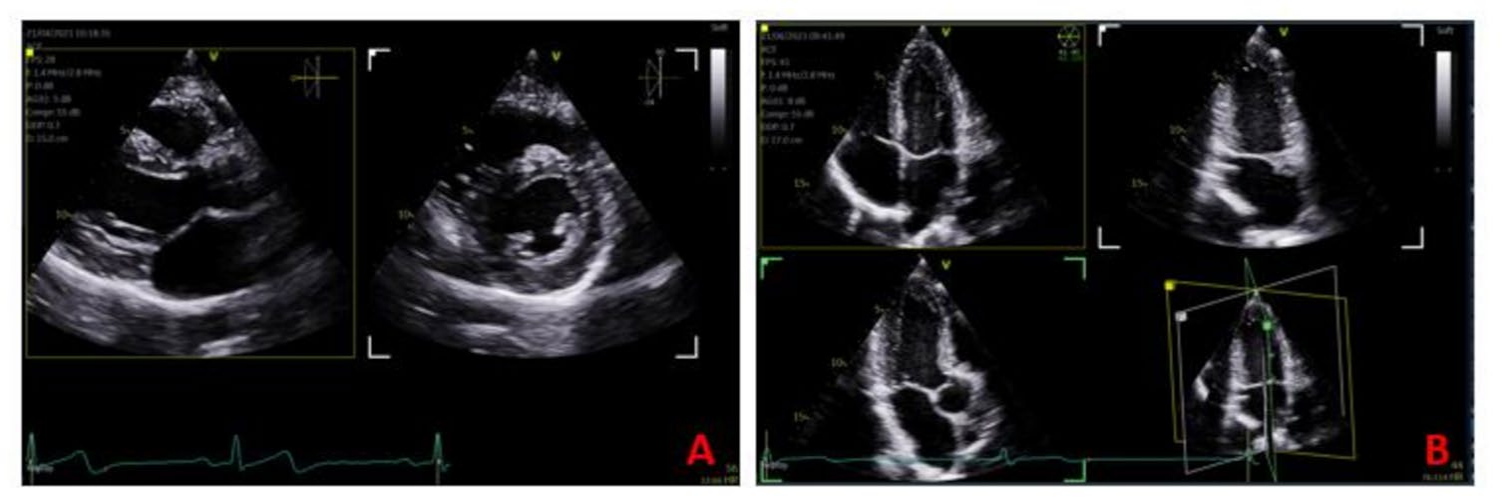
Biplane and Triplane imaging. A biplane image (**A**) of the parasternal view of the heart. The biplane cursor has been positioned at the level of the papillary muscles in the long axis view to display the corresponding short axis image on the right. The triplane image (**B**) displays the original scan plane of the apical 4 chamber view on the top left. The triplane angle has been adjusted to display the on-axis apical two chamber view (top right-hand panel) and apical long axis view (bottom left-hand panel)

**Fig. 2:**
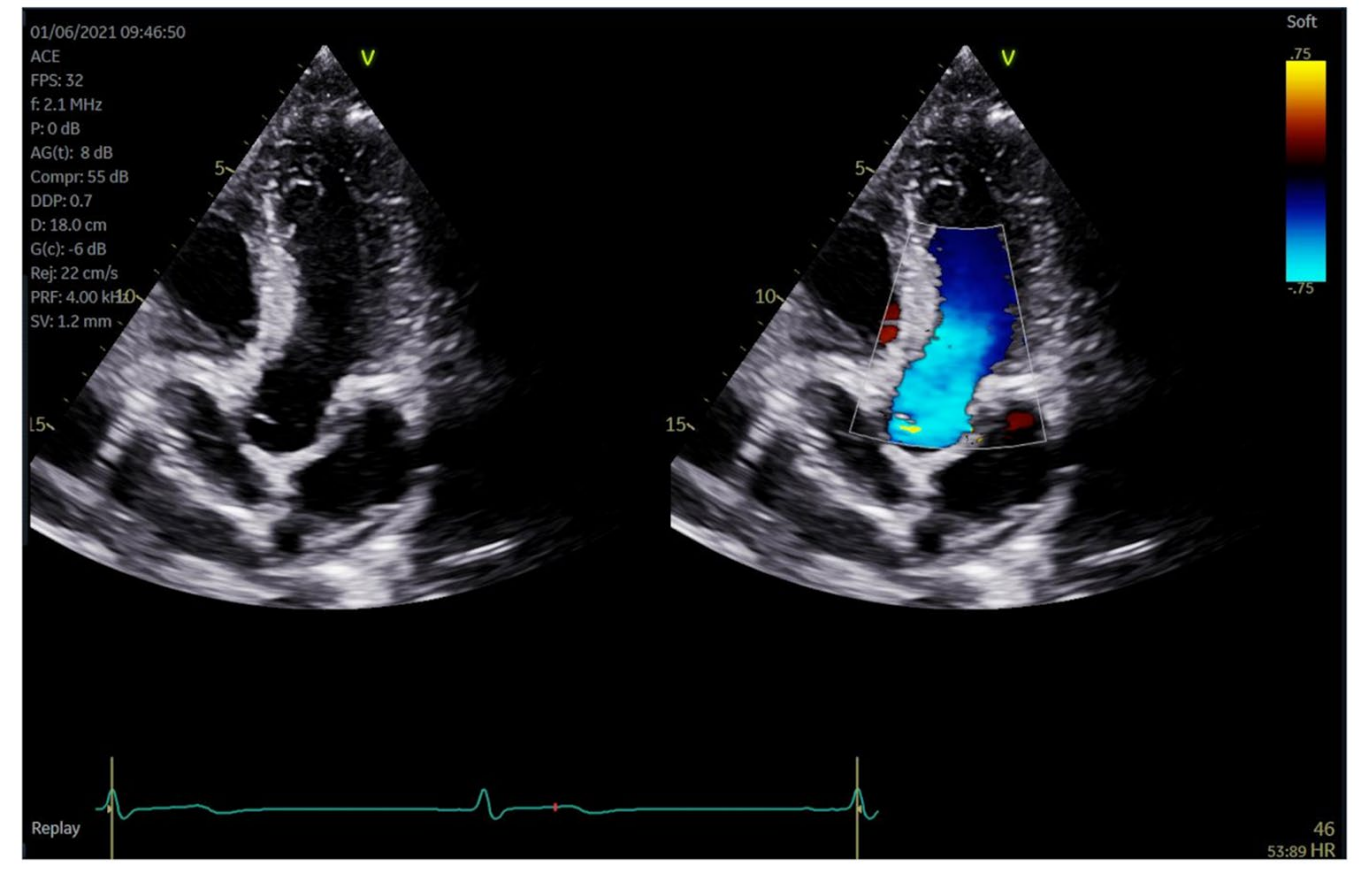
Simultaneous Colour Doppler Imaging. An apical 5 chamber view demonstrates simultaneous colour mode where both the 2D and colour Doppler images are displayed together in real time

Biplane and triplane image settings were built into the scan assistant settings for protocol A to facilitate the simultaneous acquisition of additional scan planes, reducing the number of steps required to acquire all the images for a comprehensive scan. A sweep of the cursor in biplane mode through the parasternal long axis (PLAX) structures of the heart enabled the corresponding display and capture of the short axis image (Fig. 1A). Built in triplane imaging from the apical window displayed the apical four chamber, two chamber and three chamber view in one scan assisted step (Fig. 1B). Simultaneous colour Doppler imaging built in lieu of single B mode and colour Doppler images facilitated dual viewing of the underlying 2D image at the time of colour Doppler image capture to reduce the number of images required (Fig. 2).

Commercially available quantification tools with automation and AI algorithm-based view recognition technology were built into protocol A. This technology utilised AI algorithms to recognise the underlying B mode image and imaging mode in use (2D imaging vs. continuous wave (CW) vs. pulsed wave (PW) spectral Doppler) to identify and automatically apply the correct measurement parameters, circumventing the need for manual searching and selection [11]. For example, in measurement of the left ventricular (LV) dimensions from the PLAX view, the AI auto measure 2D tool (GE Healthcare, Horten, Norway), recognizes an optimal PLAX image, selects both end diastole and end systole based on the ECG (with manual override available) and performs and displays the diastolic and systolic LV measurements in one view from one button selection. For CW and PW assessments an AI auto measure tool (Cardiac Auto Doppler, GE Healthcare, Horten, Norway) with spectrum recognition identifies the imaging mode, checks the cursor placement, identifies the position of the spectral doppler baseline and applies a cardiac auto doppler measurement if available or opens the correct measurement parameter if manual measurement is required.

On cart cardiac strain packages integrated with automatic volume, ejection fraction (EF) and emptying fraction (AFI 3.0 and Auto EF; LA strain, GE Healthcare, Horten, Norway) were utilised for automatic measurements of LV and left atrial (LA) volume. For LV assessment the three views required for LV longitudinal strain are automatically recognized based on anatomical features in the 2D image and matched based on similar heart and frame rates. The region of interest for LV EF and LA volume is automatically placed based on strain detection of endocardial borders and tracking of end diastole and end systole for the 4 chamber and 2 chamber view and available for review by clicking a tab.

### Assessment of keystrokes and scan time

Two video recorders were positioned to record both keystrokes and total scanning time. The first recording focused on the keyboard of the ultrasound machine to show the position and movement of the non-scanning hand. The second video recorded the scanning arm focusing on hand, wrist, and arm position. Scan time was measured from the time of the first TTE image until completion of the final TTE image with pauses in scanning (e.g. to reposition the participant, scanning hand, bed or adding gel to the probe for a new TTE window) removed from the calculated length of time. Post-processed measurements considered part of the standard examination were included in the calculated scan length.

Manual counting was used from the videos to collect the overall quantity of keystrokes. The keystroke actions were classified according to the specific physical activity and the location on the ultrasound console (supplementary Fig. 1). Hand, wrist, and shoulder movements were observed and categorized to assist with ergonomic assessment and graded using MODAPTS (MODodular Arrangement of Predetermined Time Standards) a predetermined motion time system (PMTS) (supplementary Table 1) [12]. Extended reach of the arm was determined by any movement that took the arm more than 30 degrees from its neutral position.

MODAPTS has been used in both industry and production research to determine and compare production times, to help design ergonomic processes, and estimate safety and quality of existing tasks [13]. This form of PMTS deconstructs all physical task actions into twenty basic human body actions, with each action allocated a standard time, based on a single time unit or MOD of 0.129 s. Human body actions are divided into a taxonomy of actions such as M: Movement, P: Puts, G: Gets, E: Eye fixation, D: Decision, K: Keying etc. Each human body action is allocated a letter and number to describe the action and associated time value– for example movement of the shoulder forward (M) is allocated 5 MOD units (5 × 0.129 s) and is described as M5– whilst movement of the elbow is allocated 4 MOD units (4 × 0.129 s) and is described as M4.

The steps required to complete each keystroke task on the ultrasound console were assessed using video data and assigned a series of “MOD actions.” The sum of these “MOD actions” then became a MOD value to assist with evaluating the performance of each task as demonstrated in supplementary Table 1.

### Assessment of image quality

The B mode and colour Doppler image quality of all participants was assessed by both senior sonographers and graded as either good, average or poor with good indicating all chambers, valves and left and right ventricular wall segments seen clearly, average indicating all chambers and valves visualised, however 1 to 2 left or right ventricular wall segments were difficult to see and poor indicating that the chambers and valves were mainly able to be visualised but more than 2 left or right ventricular wall segments were difficult to visualise.

Images from 20 randomised studies (10 from protocol A and 10 from protocol B on the same participant) were given to an expert echocardiologist for an independent assessment of image quality to determine if there was any change in image quality between study protocols. The protocol used was blinded from the reviewer by saving stored multiplane images from protocol A into a single plane for assessment.

### Statistical methods

The Statistical Package for the Social Sciences (IBMCorp. 2020) was used for data analysis. Data are expressed as mean ± standard deviation or number and percentage where appropriate. A paired samples T Test was used to compare scan time and machine interactions (quantity and MODAPTS type) recorded during each protocol. P values < 0.05 were considered statistically significant.

## Results

Thirty-five volunteer participants were recruited, totaling 70 cardiac ultrasounds. The age of the participants was 39.5 ± 16.6 years with 26 (74%) females. There were 3 (9%) participants with abnormalities detected, including mild aortic regurgitation and mild left ventricular dysfunction. The average body surface area was 1.84 ± 0.16m^2^. There were 4 (11%) participants with poor apical image quality due to either body habitus or breast augmentation. The demographic data for each participant is displayed in supplementary Table 2. 

**Fig. 3:**
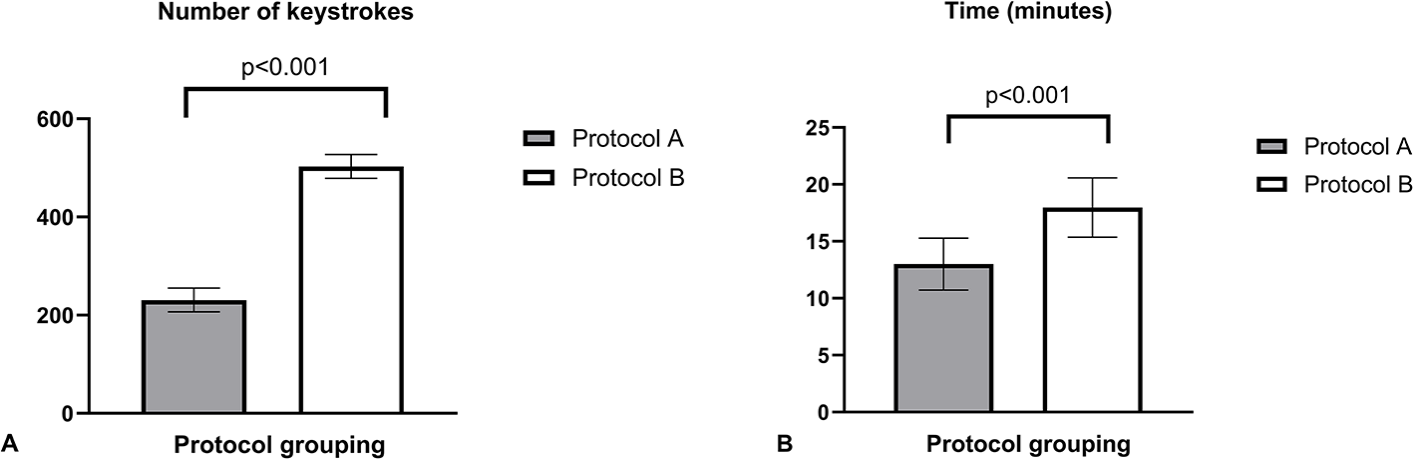
**A**. Number of console Keystrokes. **B**. TTE Scan Time (minutes). Keystroke and scan time data for protocol A vs. protocol B, showing number of keystrokes (**A**) and length of examination time in minutes (**B**) for protocol A vs. protocol B

The overall results from the statistical analysis comparing protocol A and B are discussed below. The scan time of protocol A was shorter than that of protocol B (13.4 ± 2.3 min vs. 18.0 ± 2.6 min; difference 4.6 ± 2.9 min, *p* < 0.001). Keystrokes were markedly reduced for scans performed under protocol A as compared to protocol B the standard unmodified scan (230.9 ± 24.2 vs. 502.8 ± 56.2; difference 271.9 ± 61.3, *p* < 0.001) (Fig. 3a and b).

The average keystrokes saved using a scan assistant tool, simultaneous colour Doppler display, multiplane imaging and AI are displayed in Table 1.

**Table 1:** Average modified protocol (A) keystrokes by tool used

Tool	Average Keystrokes Saved
Scan Assist Pro with built in simultaneous colour	171.5 ± 38.6
Biplane/ Triplane Imaging	16.1 ± 3.7
AI 2D PLAX Linear LV Measurements	16.3 ± 3.5
AI Doppler Measurements	27.1 ± 6.2
AFI, Auto EF	27.7 ± 5.9
LA volumes via LA Strain	13.2 ± 3.2

AFI: Automated Functional Imaging– a speckle tracking tool for assessment of myocardial strain, AI: artificial intelligence, EF: Ejection Fraction, LA: Left Atrium, PLAX: Parasternal Long Axis

An independent assessment of image quality (Table 2) from 20 random echocardiograms (10 from protocol A and 10 from protocol B) found no significant difference in the image quality and interpretability between images of a single plane origin (protocol B) and single images that had been obtained using multiplane (biplane and triplane) technology (protocol A).

**Table 2:** Image quality assessment between protocol A and protocol B

Variable	Image from protocol A	Image from protocol B
Overall parasternal quality	Good = 10 (100)	Good = 10 (100)
Overall apical quality	Good = 9 (90) Average = 1 (10)	Good = 10 (100)
Parasternal 2D imaging quality	Good = 10 (100)	Good = 10 (100)
Parasternal colour imaging quality	Good = 10 (100)	Good = 10 (100)
Apical 2D imaging quality	Good = 10 (100)	Good = 10 (100)
Apical colour imaging quality	Good = 10 (100)	Good = 10 (100)

Values are n (%) *n* = 10

Good = All chambers, valves and wall segments seen clearly

Average = 1–2 segments not seen or difficult to analyse on 2D, colour fill and quality less optimal

### MODAPTS assessment and scoring

**Fig. 4:**
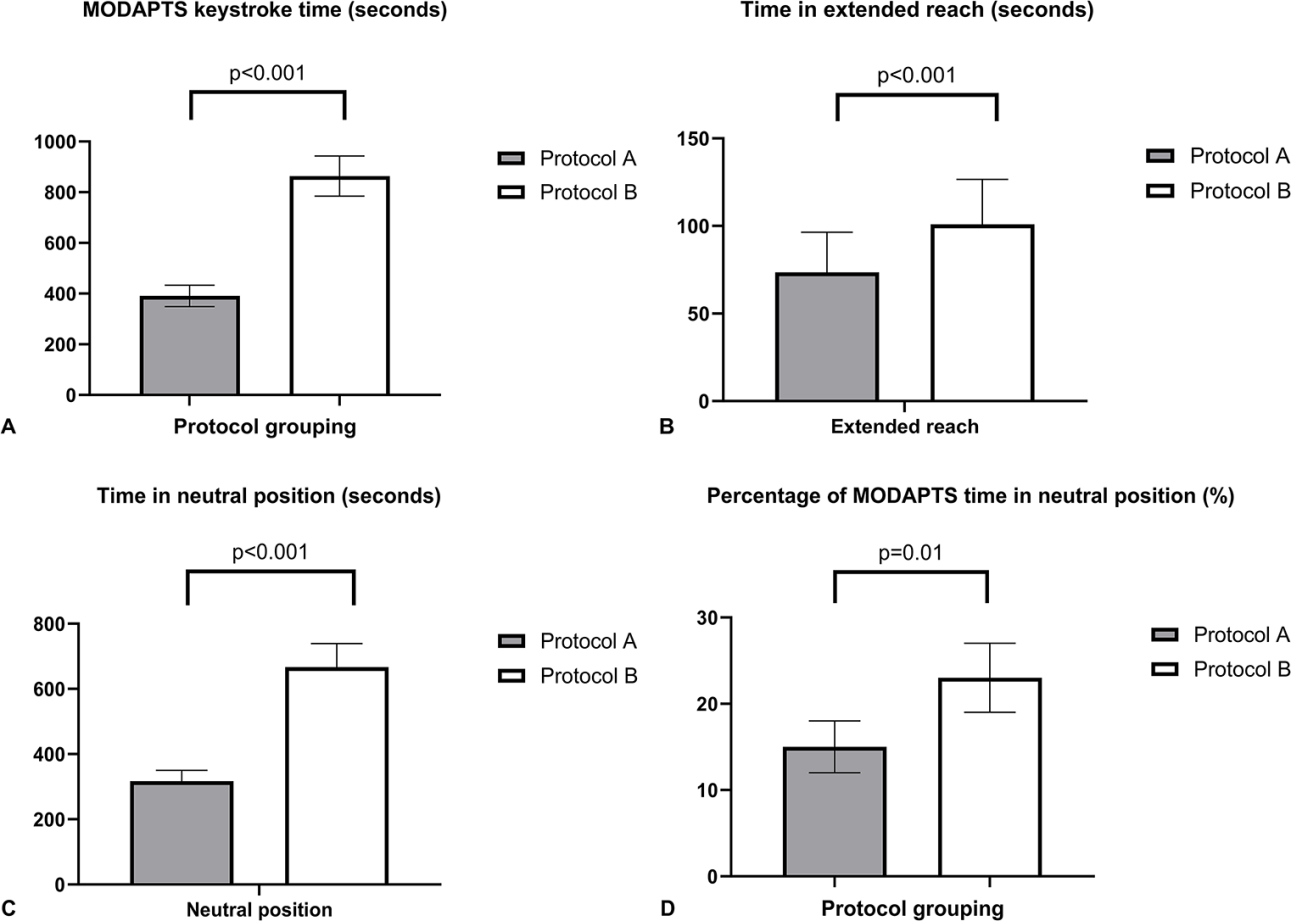
Secs = seconds. Overall MODAPTS keystroke time score between protocol A and B is demonstrated in (**A**) with a breakdown of MODAPTS time scoring for an extended reach (arm extension > 30degrees) of the arm beyond neutral on the ultrasound console (**B**) and neutral arm positioning (arm extension at < 30 degrees, **C**). The percentage of overall MODAPTS time in neutral position is demonstrated in (**D**)

There were 31 participants available for video assessment for protocol A and 30 for protocol B due to an issue with video capture. For the modified protocol (A), Sonographer 1 scanned 19 (61%) and Sonographer 2 scanned 12 (39%) participants. For the standard protocol (B), Sonographer 1 scanned 17 (55%) and Sonographer 2 scanned 13 (42%) participants. Protocol A, the modified scan resulted in a reduction in overall console movements (Fig. 4a), including the time spent with the arm in extended reach beyond 30 degrees on the console (Fig. 4b) (protocol A = 73.5 ± 22.9 s vs. protocol B = 101 ± 25.6 s, *p* < 0.001) and neutral position on the console (317 ± 33 s vs. 667 ± 72 s, *p* < 0.001) (Fig. 4c). Furthermore, the modified protocol (A) resulted in a higher percentage of the overall scan time spent in the neutral position (23 ± 4% for protocol A vs. 15 ± 3% for protocol B, *p* = 0.01), (Fig. 4d), despite an overall reduction in the length of the scan time.

## Discussions

To date there have been several studies looking at the prevalence of WRMSD in sonographers as well as studies that assess the use of technologies such as AI and automation for workflow improvement. However, to the author’s knowledge, this is the first comprehensive study comparing both while also assessing image quality and interpretability.

A modified protocol (A) not only reduced overall scan time by approximately 25% and keystrokes by > 50%, but it also demonstrated a reduction in the work and reach required as demonstrated by a reduced MODAPTS score as compared to the standard protocol (B). This reduced MODAPTS work time created by protocol A, gives opportunities for microbreaks for the shoulder through a reduction in reaching of the arm on the console beyond the neutrally placed trackball position. This allows for a more ergonomically neutral position for the elbow and shoulder with more optimal positioning of the upper limb. Reductions in reach on the console assists to align TTE with best practice recommendations to decrease ergonomic workload and reduced risk particularly for single modality sonographers such as cardiac sonographers who cannot easily follow recommendations to vary workload [5, 14].

Industry recommendations from 26 sonography-related professional organizations, accreditation bodies, and manufacturers identified multiple contributing factors to sonographer WRMSD [15]. Over-extension of the upper limbs away from neutral positioning, unnatural wrist positions and repetitive movements of the hands have been flagged as risk factors for the development of pain and discomfort. Simonsen et al. [16]. found the console arm of cardiac sonographers to be elevated beyond thirty degrees approximately 50% of the time with the wrist noted to be in awkward positions up to 46% of the examination time. Prolonged static postures such as those used in a full TTE were found to diminish blood flow to the muscle and tendons [14]. 

AI and automation for use in TTE have been commercially available for several years and shown that it is possible to reduce the number of machine interactions and to rapidly obtain multiple measurements [3]. This study has demonstrated that incorporating advanced technologies and scan assistant protocols can significantly reduce the number of keystrokes. There was a greater than 50% reduction in keystrokes noted on the ultrasound equipment for scans performed under protocol A as well as the previously noted reduction of 25% in overall ultrasound scan time.

The time saving benefits of protocol A were not as remarkable as the reduction in keystrokes when compared to protocol B. This was expected by the research team as the workflow of sonographers requires focus and includes the visual assessment of images during the scan. A sonographer must not only ensure that the images they obtain are of the highest quality but also look for unexpected pathologies that may require additional images. The mental workload would not have altered between protocol A and B, however a modified protocol such as A that reduces keystrokes and potentially discomfort may assist the sonographer to focus more on the imaging, with an ergonomic study of sonographers suggesting that sonographers become distracted by pain with impacts on image quality [17, 18].

The results of the time saved through use of AI measurement tools support industry whitepaper data [11] which claims that these tools may reduce machine clicks by up to 80% (AI Auto Measure 2d, GE Healthcare, Horten, Norway). The steps required to quantify spectral Doppler images using an AI algorithm (Auto Spectrum Recognition with Cardiac Auto Doppler, GE Healthcare, Horten, Norway) were reduced by up to 10 steps and 83% of clicks dependent on the sweep speed and parameter assessed, with the greatest time and keystroke savings noted for measurements requiring multiple traces on the system as opposed to a single caliper placement.

Research has suggested an arm abduction angle of less than 30 degrees with the shoulder of both the scanning and console arm kept in a neutral position reduces the risk of injury [5, 14]. Risk prevention and management suggestions have included considerations around ergonomics and working posture, with suggestions to limit excessive reach beyond a primary reach zone. Time is also a key factor in risk reduction for WRMSD in sonography. When deviation from neutral posture could not be avoided the recommendations shifted focus to reducing the amount of time spent in risk-producing postures [15].

It should also be noted that both sonographers involved in this study had over 20 years’ experience in TTE and have used the ultrasound machine from this study extensively clinically. Pauses between scanning for repositioning of the participant for new TTE windows were removed from the total time of both scans, this explains why these mainly normal studies were completed in a shorter period than is expected clinically. Furthermore, at the time of the acquisition of images the sonographers were not able to reduce the level of lighting as this would impair the assessment by video of the quantity and quality of keystrokes. This level of lighting impacted the ability of the sonographers to fine tune and optimize the images which would normally contribute to a longer scan time. At the time of this study, some of the new AI tools now available were not able to be used on cart, including a fully automated LV quantification package (Easy AFI, GE Healthcare, Horten, Norway). With modern technologies, this difference in time may be further increased, but more research is required.

An independent assessment of images obtained performed during this study found that Protocol A showed no significant difference in quality as compared to the single plane images and manually performed measurements from Protocol B. Images from both protocols were deemed fully analysable.

There are several limitations to this project. This was a single centre study with a small sample size. Most participants were young, normal volunteers without a history of heart disease. This technology should be validated in various cardiac pathologies to demonstrate its time and keystroke saving potential in other populations. Furthermore, this study was focused on overall time saving and workload reduction. It did not directly compare the accuracy and reproducibility of the AI tools used. Further research comparing these tools will provide greater insight into their direct benefit and the reproducibility and accuracy of the measured results they produce.

Unfortunately to date, AI and Automation, including semi-automated scan assistant protocols, are not uniformly available with all echocardiography vendors. Many echocardiography departments also do not have affordable access to 4D capable “all in one” cardiac imaging probes limiting widespread use of this technology to assist with cardiac sonographer workflow. Future research to further investigate the use of commercially available technologies on a variety of platforms is recommended and will also assist in addressing the above limitations.

Despite the limitations noted, this research has highlighted the potential keystroke and time-saving benefits for cardiac sonographers and the potential for a reduction in risk of WRMSD. A more complete assessment of the difference in physical workload for sonographers between the two protocols would be required to ascertain the full ergonomic benefit of a modified scan protocol.

## Conclusion

This study indicates: (1) There was a reduced acquisition and processing time for a modified protocol compared with a full standard echocardiogram and (2) There was a significant reduction in operator keystrokes and reaching beyond a recommended neutral position for a modified protocol as compared with a full standard protocol echocardiogram without a reduction in overall quality and interpretability.

The results support the implementation of multiplane imaging, scan assistant protocols and semi/ fully automatic AI technology during image acquisition to reduce sonographer scan time, keystrokes and shows potential for improved sonographer ergonomics.

## Electronic supplementary material

Below is the link to the electronic supplementary material.

Supplementary Material 1

## Data Availability

The data that support the findings of this study are available from University of South Australia but restrictions apply to the availability of these data, which were used under license for the current study, and so are not publicly available. Data are however available from the authors upon reasonable request and with permission of University of South Australia.

## Abbreviations

2D: Two dimensional
4D: Four dimensional
AFI: Automated functional imaging
AI: Artificial intelligence
ASE: American society of echocardiography
EF: Ejection fraction
LA: Left atrium
LV: Left ventricular
MODAPTS: Modular arrangement of time standards
PLAX: Parasternal long axis
PMTS: Predetermined motion time system
TTE: Transthoracic echocardiogram
WRMSD: Work related musculoskeletal disorders
