# Supplementary material for: Can automation and artificial intelligence reduce echocardiography scan time and ultrasound system interaction?
Source: Echo Res Pract. 2025 Jun 16;12:11. doi: 10.1186/s44156-025-00077-0 (PMC12168272; doi:10.1186/s44156-025-00077-0)
Supplement: Supplementary file 1 — Supplementary Material 1 [file 44156_2025_77_MOESM1_ESM.docx]

**Supplementary Figures and Tables**

| 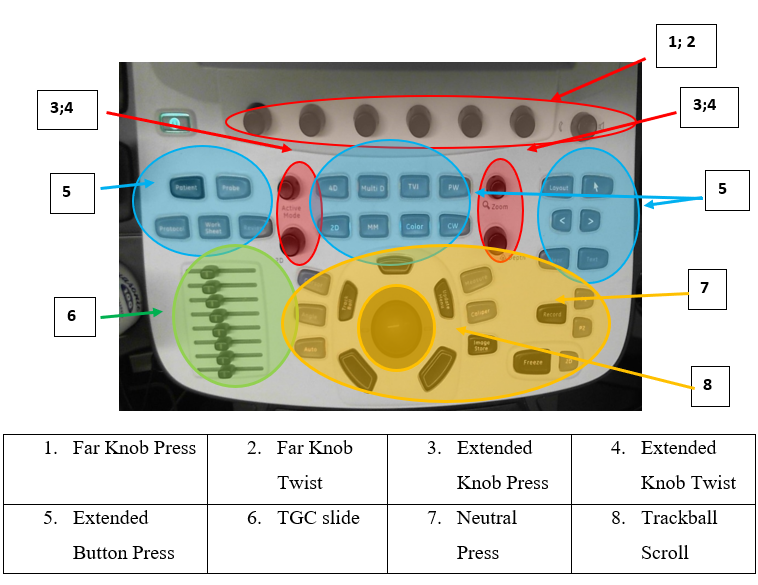 |
| --- |
| Supplementary figure 1: Ultrasound console grouping based on movement from neutral position and nature of movement. |

Supplementary table 1: MODAPTS coding of each grouped ultrasound console interaction.

| Console Interaction | Movement Description for MOD scoring | MOD Actions | MOD Value |
| --- | --- | --- | --- |
| Trackball Scroll | Movement of hand (hinged at wrist (M2)), simple contact (no grip (G0)), puts with feedback (P5), eye fixation (no movement of gaze (E2)) and simple binary decision (D3)) | M2, G0, P5, E2, D3 | 12 |
| Neutral Press | Movement of hand (hinged at wrist (M2)), simple contact (no grip (G0)), simple put (put with feedback (P2), eye fixation (no movement of gaze (E2)), simple binary decision (D3)) and routine key (little resistance (K1.5)) | M2, G0, P2, D3, E2, K1.5, D3 | 13.5 |
| Central Extend Press | Movement of forearm (hinged at elbow (M3)), simple contact (no grip (G0)), simple put (put with feedback (P2), eye fixation (no movement of gaze (E2)), simple binary decision (D3)) and routine key (little resistance (K1.5)) | M3, GO, P2, E2, D3, K1.5 | 11.5 |
| R/L Extend Knob Press | Movement of forearm (hinged at elbow (M3)), simple contact (no grip (G0)), simple put (put with feedback (P2), eye fixation (no movement of gaze (E2)), simple binary decision (D3)) and routine key (little resistance (K1.5)) | M3, GO, P2, E2, D3, K1.5 | 11.5 |
| Central Far Knob Press | Movement of arm (hinged at shoulder (M4)), simple contact (no grip (G0)), simple put (put with feedback (P2), eye fixation (no movement of gaze (E2)), simple binary decision (D3)) and routine key (little resistance (K1.5)) | M4, GO, P2, E2, D3, K1.5 | 12.5 |
| TGC Slide | Movement of forearm (hinged at elbow (M3)), simple contact (no grip (G0)), puts with feedback (P5) eye fixation (no movement of gaze (E2)), simple binary decision (D3)) and routine key (little resistance (K1.5)) | M3, G0, P5, E2 | 10 |
| R/L Extend Knob Twist | Movement of forearm (hinged at elbow (M3)) grip with feedback (G3), puts with feedback (P5), eye fixation (no movement of gaze (E2)) and simple binary decision (D3)) | M3, G3, P5, E2, D3 | 16 |
| Central Far Knob Twist | Movement of arm (hinged at shoulder (M4)), grip with feedback (G3), puts with feedback (P5), eye fixation (no movement of gaze (E2)) and simple binary decision (D3)) | M4, G3, P2, E2, D3 | 17 |

Supplementary Table 2: Study population n = 35

| **Demographics** | n = 35 |
| --- | --- |
| Age (yrs) | 43 +/- 16 |
| Female | 26 (74) |
| Hx IHD | 1 (3) |
| Smoker | 3 (9) |
| High cholesterol | 1 (3) |
| Hypertension | 2 (6) |
| FHx premature CVD | 1 (3) |
| **Clinical characteristics** |  |
| BSA (m2) | 1.83 +/- 0.16 |
| **Parasternal Image quality** |  |
| Poor (>2 segments missing) | 0 |
| Fair (1-2 segments missing) | 4 (13) |
| Good (0 segments missing) | 31 (87) |
| **Apical Image Quality** |  |
| Poor (>2 segments missing) | 4 (11) |
| Fair (1-2 segments missing) | 2 (6) |
| Good (0 segments missing) | 29 (83) |
| Values are n (%) or mean =/- SD (range)  BSA= Body surface area. | |

Supplementary Table 3 : Research Scan Protocol A

AI/4D echo protocol – programmed into ScanAssist Pro (automatically changes depth, colour scale, Doppler scale and puts on colour, zoom or Doppler cursor)

| **Research Scan Protocol A** |  |
| --- | --- |
| Parasternal LAX: |  |
| • 2D with deep FOV to exclude pericardial/ pleural effusion |  |
|  |  |
| • Biplane clip with SAX at level of apex |  |
|  |  |
| • Biplane clip with SAX at level of papillary muscles |  |
|  |  |
| • Biplane clip with SAX at level of MV |  |
|  |  |
| • Biplane clip with CFI on MV |  |
|  |  |
| • Biplane clip with SAX at level of AoV |  |
|  |  |
| • Biplane clip with CFI on AV |  |
|  |  |
| • Zoom on AV to measure LVOT diameter (manual) |  |
|  |  |
| • PLAX clip for 2D auto-measure (AI measurement) |  |
|  |  |
| • Ascending aorta in real time |  |
|  |  |
| • Ascending aorta 2D measurement (manual) |  |
|  |  |
| • RV inflow tract with simultaneous CFI |  |
|  |  |
| • CW through TV (auto-measure TR Vmax if applicable) |  |
|  |  |
| • RV outflow tract with simultaneous CFI |  |
|  |  |
| • CW through PV |  |
|  |  |
| Parasternal SAX: |  |
|  |  |
| • View of IAS with simultaneous CFI |  |
|  |  |
| • View of TV with simultaneous CFI |  |
|  |  |
| • CW through TV (auto-measure TR Vmax if applicable) |  |
|  |  |
| Apical 4ch: |  |
|  |  |
| • Triplane image of all 3 apical views – full depth |  |
|  |  |
| • Triplane image of all 3 apical views – LV focus |  |
|  |  |
| • Triplane with CFI over MV |  |
|  |  |
| • CW though MV |  |
|  |  |
| • PW MV inflow |  |
|  |  |
| • Auto-measure: E & A velocity, DT and A duration |  |
|  |  |
| • PW pulmonary veins |  |
|  |  |
| • Auto-measure: S, D, AR measurements and AR duration |  |
|  |  |
| • Doppler tissue imaging |  |
|  |  |
| • PW Mitral Septal annulus |  |
|  |  |
| • Auto-measure septal E’ |  |
|  |  |
| • PW Mitral Lateral annulus |  |
|  |  |
| • Auto-measure E’ |  |
|  |  |
| • Right heart optimised view with simultaneous CFI |  |
|  |  |
| • CW through TV (auto-measure TR Vmax if applicable) |  |
|  |  |
| • Measure RAV (manual) |  |
|  |  |
| • Reduced depth view for RV measurements (manual) |  |
|  |  |
| • TAPSE (manual) |  |
|  |  |
| • RV S’ (manual) |  |
|  |  |
| • LV focussed view for 4ch AFI (auto-measure after acquisition of apical LAX view) |  |
|  |  |
| • LA focussed view for 4ch LA AFI (semi-automated measure after acquisition of apical 2ch view) |  |
|  |  |
| • 4D full volume (all 4 chambers) |  |
|  |  |
| • Auto-measure LVEF and LAV from 4D |  |
|  |  |
| Apical 5ch view |  |
|  |  |
| • 2D clip with simultaneous CFI over AV |  |
|  |  |
| • PW LVOT |  |
|  |  |
| • Auto-measure LVOT VTI |  |
|  |  |
| • CW AV |  |
|  |  |
| • Auto-measure AV VTI |  |
|  |  |
| Apical 2ch view |  |
|  |  |
| • LA focussed view for 2ch LA AFI (measure LA strain and volumes using LA AFI package – semi-automated) |  |
|  |  |
| • LV focussed view for 2ch AFI (auto-measure after acquisition of apical LAX view) |  |
|  |  |
| Apical LAX view |  |
|  |  |
| • LV focussed view for apical LAX AFI |  |
|  |  |
| • Auto-measure for LVEF and GLS |  |
|  |  |
| Subcostal view |  |
|  |  |
| • Clip of all 4ch with simultaneous CFI over IAS |  |
|  |  |
| • SAX images at all levels |  |
|  |  |
| • IVC view |  |
|  |  |
| • M-mode for size and reactivity |  |
|  |  |
| • CFI of IVC/ hepatic vein |  |
|  |  |
| • PW hepatic vein |  |
|  |  |
| Suprasternal view |  |
|  |  |
| • 2D clip |  |
|  |  |
| • CFI descending aorta |  |
|  |  |
| • PW +/- CW in descending aorta |  |
|  |  |

Supplementary Table 4: Scan Protocol B. Full manual echo protocol

| Scan Protocol B. Full echo protocol |  |
| --- | --- |
|  |  |
| Parasternal Long Axis (PLAX) view: |  |
|  |  |
| • 2D with deep field of view to exclude pericardial/ pleural effusion |  |
|  |  |
| • 2D at best depth for PLAX view |  |
|  |  |
| • 2D measurement of LV |  |
|  |  |
| • Zoom AV |  |
|  |  |
| • LV outflow tract (LVOT) diameter measurements (at least 2) |  |
|  |  |
| • Colour flow imaging (CFI) over AV both on and off zoom (pan through valve) |  |
|  |  |
| • Zoom mitral valve (MV) |  |
|  |  |
| • Colour flow imaging over MV both on and off zoom (pan through valve) |  |
|  |  |
| • Ascending aorta clip in real time |  |
|  |  |
| • Ascending aorta 2D measurement |  |
|  |  |
| Parasternal LAX right ventricular (RV) inflow view: |  |
|  |  |
| • 2D clip |  |
|  |  |
| • CFI clip over tricuspid valve (TV) (pan through valve) |  |
|  |  |
| • Continuous wave Doppler (CW) through tricuspid regurgitation (TR) jet if present + measure peak velocity |  |
|  |  |
| PLAX RV outflow tract (RVOT): |  |
|  |  |
| • 2D clip |  |
|  |  |
| • CFI clip over pulmonary valve (PV) (pan through valve) |  |
|  |  |
| • CW across PV + measure |  |
|  |  |
| • Pulsed wave Doppler (PW) of RVOT + measure |  |
|  |  |
| Parasternal short axis (PSAX) AV level: |  |
|  |  |
| • 2D clip |  |
|  |  |
| • AV zoom |  |
|  |  |
| • CFI over AV (pan through valve) |  |
|  |  |
| • 2D pulmonary artery (PA) bifurcation |  |
|  |  |
| • CFI RVOT/PA bifurcation (pan through to look for acceleration of flow) |  |
|  |  |
| • CW across PV + measure |  |
|  |  |
| • PW of RVOT + measure |  |
|  |  |
| • CFI wider across AV (to rule out ventricular septal defect) |  |
|  |  |
| • CFI TV (pan through valve) |  |
|  |  |
| • CW if TR present + measure |  |
|  |  |
| • CFI interatrial septum (IAS) (reduce colour scale and pan through IAS) |  |
|  |  |
| PSAX MV level: |  |
|  |  |
| • 2D clip |  |
|  |  |
| • MV zoom |  |
|  |  |
| • CFI MV (pan through valve) |  |
|  |  |
| PSAX pap muscle level |  |
|  |  |
| • 2D clip |  |
|  |  |
| PSAX LV apex |  |
|  |  |
| • 2D clip |  |
|  |  |
| Apical 4ch: |  |
|  |  |
| • 2D (all 4 chambers) |  |
|  |  |
| • Optimise for LA volume and measure |  |
|  |  |
| • Zoom MV |  |
|  |  |
| • CFI MV both zoom on and off (pan through valve) |  |
|  |  |
| • CW if MR present |  |
|  |  |
| • PW MV inflow |  |
|  |  |
| • Measurements: E & A velocity, DT and A duration |  |
|  |  |
| • CFI pulmonary veins |  |
|  |  |
| • PW pulmonary veins |  |
|  |  |
| • S, D, AR measurements and AR duration |  |
|  |  |
| • Doppler tissue imaging |  |
|  |  |
| • Septal annulus + measurement E’ |  |
|  |  |
| • Lateral annulus + measurement E’ |  |
|  |  |
| • Optimised LV view |  |
|  |  |
| • Simpson’s biplane |  |
|  |  |
| • RV/RA optimised view |  |
|  |  |
| • RAV |  |
|  |  |
| • CFI TV (pan through valve) |  |
|  |  |
| • CW if TR present + measure |  |
|  |  |
| • TAPSE + RV s’ |  |
|  |  |
| • RV optimised view |  |
|  |  |
| • RV basal, mid and length |  |
|  |  |
| • 3D volume acquisition for LVEF and LAV |  |
|  |  |
| Apical 5ch view |  |
|  |  |
| • 2D clip |  |
|  |  |
| • CFI AV (pan though valve) |  |
|  |  |
| • PW LVOT |  |
|  |  |
| • Measure LVOT VTI |  |
|  |  |
| • CW AV |  |
|  |  |
| Apical 2ch view |  |
|  |  |
| • 2D clip of LV/LA |  |
|  |  |
| • LA volume |  |
|  |  |
| • Zoom MV |  |
|  |  |
| • CFI MV (pan through valve) |  |
|  |  |
| • CW of MR if present |  |
|  |  |
| • Optimised view for LV |  |
|  |  |
| • Simpson’s biplane |  |
|  |  |
| Apical LAX view |  |
|  |  |
| • 2D clip of LV/LA/Ao |  |
|  |  |
| • Zoom AV |  |
|  |  |
| • CFI AV (pan though valve) |  |
|  |  |
| • Zoom MV |  |
|  |  |
| • CFI MV (pan through valve) |  |
|  |  |
| • Optimised view for LV |  |
|  |  |
| • Perform LV global longitudinal strain measurement (manual tracking, manual selection of three apical views) |  |
|  |  |
| • Perform LA strain measurement (manual tracking) |  |
|  |  |
| Subcostal view |  |
|  |  |
| • Clip of all 4ch |  |
|  |  |
| • CFI over IAS (pan though) |  |
|  |  |
| • CFI over IVS (pan through) |  |
|  |  |
| • SAX images at all levels |  |
|  |  |
| • Inferior vena cava (IVC) with ‘sniff’ in 2D |  |
|  |  |
| • M-mode for size and reactivity (with ‘sniff’) |  |
|  |  |
| • CFI of IVC/ hepatic vein |  |
|  |  |
| • PW hepatic vein |  |
|  |  |
| Suprasternal view |  |
|  |  |
| • 2D clip |  |
|  |  |
| • CFI descending aorta |  |
|  |  |
| • PW +/- CW in descending aorta |  |
|  |  |
